# Severe maternal morbidity in the high income setting: a systematic review of composite definitions

**DOI:** 10.1016/j.eclinm.2025.103105

**Published:** 2025-02-13

**Authors:** Ian Henderson, Rosie Lynch, Stephen Gerry, Jenny McLeish, Peter Watkinson, Marian Knight

**Affiliations:** a National Perinatal Epidemiology Unit, Nuffield Department of Population Health, University of Oxford, Oxford, OX3 7LF, UK; b Centre for Statistics in Medicine, Nuffield Department of Orthopaedics, Rheumatology and Musculoskeletal Sciences, University of Oxford, Oxford, OX3 7LD, UK; c Critical Care Research Group, Nuffield Department of Clinical Neurosciences, University of Oxford, Oxford, UK

**Keywords:** Severe maternal morbidity, Morbidity, Pregnancy, Pregnancy complications, Outcome definition, Maternal health

## Abstract

**Background:**

Severe maternal morbidity (SMM) is an important indicator for the improvement of maternity care. Measurement of SMM varies, limiting global comparisons. To promote concordance we studied how SMM has been defined in epidemiological practice.

**Methods:**

Comprehensive composite definitions of SMM in pregnancy or up to 6 weeks postnatal that captured both obstetric and non-obstetric processes in high-income settings were identified through a prospectively registered (PROSPERO CRD42023421377) systematic search of PubMed, Embase, and Google Scholar 01/01/1993–31/08/2024. Clinical concepts, diagnostic and procedural codes captured by definitions of SMM were compared and the variation between definitions was described.

**Findings:**

The initial search identified 7852 records and 40 studies were included: 28 studies that reported 32 definitions of SMM for use with administrative data, with median incidence of 11.4/1000, and 13 studies that reported 13 definitions for use with the primary medical record, with median SMM incidence of 6.7/1000. The majority of definitions included cardiac, respiratory, and renal dysfunction or failure; haemorrhagic, thrombotic or infective morbidity; and critical interventions. Up to 75% of cases of SMM under some definitions involved transfusion. The main source of variation between definitions was the selection and definition of common obstetric diagnoses. Variation in the sources of additional routine data required to construct a definition also limited comparability.

**Interpretation:**

Despite common approaches to defining SMM, there are opportunities to improve comparability. No two definitions for use with administrative data in different settings involved a similar incidence and set of components and involved a similar distribution of components among cases. Harmonization of the purpose, constituent codes, and sources of data would facilitate comparisons between maternity systems.

**Funding:**

This work was supported by the 10.13039/501100000265Medical Research Council [MR/X006115/1] as well as the 10.13039/501100000272National Institute for Health Research [NIHR204430].

**Research in context**

**Evidence before this study**

Severe maternal morbidity is recognised as a key outcome for surveillance outcome and indicator for the purpose of quality improvement. The World Health Organisation defined severe maternal morbidity based on clinical, laboratory, and management criteria but this definition is not readily applicable to either routine administrative or clinical data and is challenging to scale to a national or international level. Multiple definitions of severe maternal morbidity have therefore been developed but the relationship between definitions had not been described.

**Added value of this study**

Among definitions for use with administrative data we found that diagnostic codes for the concepts of end-organ injury and critical interventions were most consistently included, whereas the inclusion of diagnoses of severe obstetric morbidity varied. We found that composite measures often included individual components that differed in incidence, severity, the proportion of cases they represent, and in meaning.

**Implications of all the available evidence**

The interpretation of composite severe maternal morbidity outcomes requires consideration of the constituent diagnoses, processes, and procedures in relation to its clinical context. Stakeholders who use these outcomes need to assess whether an individual outcome is appropriate for their purpose. The ability to compare severe maternal morbidity between healthcare systems, including international comparisons, would be valuable. Developing consensus around the common components involving end-organ injury and critical interventions is easily achievable; further work could also develop consensus around the less consistently included components. There is a need to develop consensus around the purpose and scope of an international definition of severe maternal morbidity using administrative health data, including consideration of whether incident disease is modifiable.

## Introduction

Maternal mortality is a very rare event in high income countries (HICs), contributed to by direct (obstetric) and indirect (non-obstetric conditions exacerbated by pregnancy) disease processes in comparable measure.^1^ Efforts to reduce maternal mortality are complicated by its rarity. Severe maternal morbidity (SMM) is more common and can capture disease processes that are precursors of maternal mortality. Reducing SMM is therefore a second quality improvement target. SMM surveillance may help to understand the conditions and processes that lead to maternal mortality more comprehensively to aid in prevention of maternal mortality. The World Health Organisation (WHO) Maternal Morbidity Working Group defined maternal morbidity as “any health condition attributed to and/or complicating pregnancy and childbirth that has a negative impact on the woman's wellbeing and/or functioning”.^2^ A continuum from maternal morbidity, through potentially life-threatening conditions, life-threatening complications, to maternal near-miss morbidity (or maternal death) has been conceptualised.^3^^,^^4^ SMM has been defined and informed by the degree of intervention, acuity, or end-organ injury.^5^ Different definitions of SMM have been developed but their coverage of the continuum outlined by the WHO differs.

Expanding the scope of surveillance to incorporate a wide range of severe morbidities means we have the ability to consider health inequalities,^6^ the prediction of SMM,^7^ causal pathways and modifiable determinants of SMM,^8^ and recommendations to improve care.^9^^,^^10^ The American College of Obstetricians and Gynecologists recommends case reviews of SMM to identify potential avoidability.^11^ To undertake these activities, conceptual definitions of SMM have to be translated into useable epidemiological measures.^12^ This process may be influenced by the setting, purpose of the definition, source of data, and the clinical or research systems within which the process occurs. Different definitions of SMM may therefore be irreconcilable, depriving quality improvement stakeholders of valuable comparisons that could identify safer practices and processes. International comparisons of SMM have the potential to guide national quality improvement^13^ but there are additional challenges when comparing SMM between countries. Definitions commonly capture major obstetric, medical, or surgical complications of pregnancy, birth, and the puerperium. There is no unified definition of SMM^14^, ^15^, ^16^, ^17^ for use with diagnostic and procedural codes found in administrative data that is scalable to an international level. Even when using similar definitions, the reliability of administrative data and coding practices may vary between settings.^18^^,^^19^ Comparisons between countries have typically relied on a narrower range of indicators more suitable for coordinated collection across surveillance systems.^20^^,^^21^

The rationale for this systematic review is that comparing ‘comprehensive’ definitions (those that incorporate both direct and indirect processes) of SMM will identify opportunities to strengthen its measurement, and that this can inform the harmonisation of approaches to facilitate quality improvement in maternity care. The aim was to understand how SMM is operationalised in HICs. The review was restricted to HICs as the incidence of SMM, availability of data, and purpose of SMM definitions may differ in other economic contexts.

## Methods

A prospectively-registered (PROSPERO CRD42023421377) systematic review of definitions of SMM was conducted. The conduct and report of this research was based on the Preferred Reporting Items for Systematic Reviews and Meta-Analyses (PRISMA) statement.^22^ Preliminary searches identified relevant terms to inform the search strategy, reported in Supplementary Table S1. The search strategy was developed with assistance from a research librarian with expertise in women's health. Records were identified through PubMed, Embase, and Google Scholar that were published between 01/01/1993 and 31/08/2024. The first 1000 records identified in Google Scholar were screened. Grey-literature searches were conducted using Bielefeld Academic Search Engine, Jisc, and OpenGrey. Observational or interventional studies in HICs that developed a unique definition of SMM and that incorporated both direct and indirect measures of SMM were included. SMM was defined as a composite outcome of at least two diagnoses, measures, or processes reflective of SMM that occurred during pregnancy and up to 6 weeks following birth. The time period is in accordance with the WHO definition of maternal near-miss.^23^ Morbidity and mortality that occurs after the postnatal period is less likely to be pregnancy-related and is driven by different processes.^24^ Definitions that included a non-severe component (i.e., a non life-threatening condition in pregnancy, such as gestational diabetes, compared with a potentially life-threatening condition^25^) or a component conditional on a specific exposure (e.g., the inclusion of ‘postoperative ventilation’ but not ‘ventilation’) were excluded. No studies were excluded based on language. Definitions of only/predominantly direct measures of SMM were excluded, as this limits their application for the prevention of SMM and maternal mortality for all causes. For example, a definition comprised of eclampsia, severe haemorrhage, and sepsis only would have been excluded. Screening, full-text reviews and systematic citation searches were conducted in duplicate by two reviewers (IH and RL). Data were extracted using a piloted data collection form using Covidence. Disagreement between reviewers was resolved by discussion.

The data were summarised using counts and percentages and a narrative synthesis was conducted, providing description and comparison of the identified measures. Study characteristics, narrative summaries of the approach to SMM development, and components of SMM definitions were reported. Definitions were grouped according to their intended use with International Classification of Disease codes^26^ found in administrative data or based on the interpretation of clinical data found in the primary medical record. The constituent diagnostic and procedural codes within clinical concepts across definitions for use with administrative data from different settings (Australia, Canada, England, Finland, Sweden, USA) were compared. A formal risk of bias assessment was not conducted for this systematic review of outcome definitions because there are no established standards for the development of a composite outcome; instead, the development of outcome definitions was described. Missing data on definitions were acquired from associated publications from the same authors. This review is reported according to the PRISMA checklist as shown in Supplementary Table S2.

### Role of funding source

No funder had any involvement in the design, conduct, analysis, interpretation, or writing of this research or the decision to submit it for publication.

## Results

**Fig. 1:**
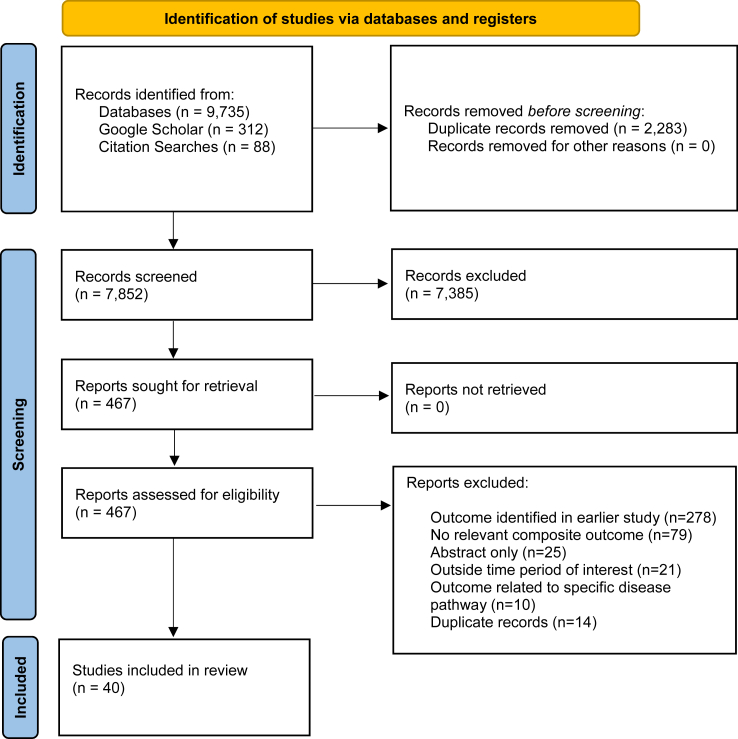
**PRISMA flow diagram**. PRISMA flow diagram outlining the study selection process. From: Page MJ et al.^22^ For more information, visit: http://www.prisma-statement.org/.

The search identified 7852 unique records and 467 were retrieved for full text review. Of these, 278 studies used a composite outcome already identified; 79 did not include a relevant composite outcome; 25 related to abstracts only; 21 occurred outside the time period of interest; 10 developed composite outcomes for specific disease pathways; and 14 duplicate were records. There were 40 studies included in total, with 28 studies that identified 32 composite definitions of SMM developed for use with routine administrative data, reported in Table 1, and 13 studies that identified 13 composite definitions for use with/abstraction from the primary medical record, reported in Table 2. The PRISMA flow diagram is shown in Fig. 1.

**Table 1:** Definitions of SMM developed for use with routine administrative data.

Definition	Data	Selection of components	Description	Incidence (/1000)
Wen 2005^27^	DD (Canada), ICD-9	“[N]ear miss conditions with potential to cause maternal death” reliably identifiable from discharge data; case fatality rates compared with reference rates.	**Cardiac arrest, HF, or cerebral anoxia after obstetric surgery (combined 24%)**, pulmonary oedema, MI, RF, ARDS, ARF, stroke, VST, **CoA (11%)**; eclampsia, PE, **uterine rupture (17%)**; **haemorrhage with transfusion (21%)** or with hysterectomy, ventilation.	Del 4.4 (1991–2001)
Olive 2005^28^	Universal perinatal database, DD (NSW, Australia)	Unspecified development of ‘major maternal morbidity’ outcome with respect to an exposure of placenta praevia.	ARF, ventilation, shock, death; **PPH with transfusion (63%), DIC, transfusion of platelets/coag. factors/FFP (combined 12%)**, hysterectomy, surgical management of haemorrhage, **ITU (21%)**.	11.2 (1998–2002)
CDC, Callaghan 2008^29^	NHDS (USA), ICD-9-CM	Informed by near-miss/SMM case review in Geller 2002^4^ and SMM in Wen 2005^27^; internal validation with respect to length of stay.	Original CDC list comprised of cardiac arrest, MI, HF (including cardiogenic pulmonary oedema), cardiomyopathy, RF, ARF, LD, stroke, VST, CoA; **eclampsia (14%)**, AFE, PE, shock, septicaemia; CPR/cardioversion, ventilation, **hysterectomy (12%)**, invasive monitoring, and **transfusion (whole blood or red cells, 48%)**. Diagnostic codes qualified by length of stay ≥3 days.	Del 5.1 (1991–2003)
AMMOI, Roberts 2008^17^	DD (NSW, Australia)	Literature review and consultation. Codes refined by comparison with case review of clinically-validated SMM, informed by availability/reliability of DD.	Cardiac arrest, HF, pulmonary oedema, MI, SA, ARF, stroke, SE, coma, acute psychosis, shock, peritonitis, appendicitis, sickle crisis, CoA; AFE, PE, DIC, uterine rupture; CPR/cardioversion, ventilation, hysterectomy, surgical management of haemorrhage, evacuation of haematoma, repair of organs, repair of inverted uterus, reclosure of CS wound, dialysis, **transfusion (any product, 75%; from Roberts 2009).**^30^	Del 14.6 (2002), Roberts 2008; del 12.5 (1999–2004), Roberts 2009^30^
Kuklina 2009^31^	NIS (USA), ICD-9-CM	Informed by WHO SMM (Say 2004) and non-composite indicators of SMM identified in Kuklina 2008.^32^	Cardiac arrest, HF, pulmonary oedema, RF, ARDS, ARF, stroke, VST, shock, CoA; DVT, PE, septicaemia and sepsis, DIC; ventilation, hysterectomy, **transfusion (56%)**.	Del 8.1 (2004–05)
CPSS, Joseph 2010^33^	DD (Canada), ICD-10CA	From Wen 2005,^27^ supplemented with additional codes/compared with Roberts 2008.^17^	As per Wen 2005 with addition of pre-existing hypertensive cardiac disease, cardiomyopathy, SA, LD, SE, coma, acute psychosis, shock, peritonitis, appendicitis, sickle crisis, HIV disease, death; AFE, sepsis/septicaemia; APH/IPH with coag. defect, DIC; CPR/cardioversion, surgical management of haemorrhage, evacuation of haematoma, repair of organ injury, dialysis, **transfusion (whole blood/red cells, 47%)**.	Del 13.8 (2003–07)
Mhyre 2011^34^	NIS (USA), ICD-9 CM	Review of literature^27^^,^^29^^,^^31^ and ICD-9 CM manual for severe, life-threatening complications reflecting end-organ injury-based definition; association of components with inpatient death or discharge to medical facility considered.	**Cardiac arrest or HF (19% combined)**, pulmonary oedema, MI, RF, **ARDS (20%)**, SA, **ARF (13%)**, **LD (21%)**, stroke, VST, SE, coma, delirium, shock, DKA, major transfusion reaction, panhypopituitary syndrome, CoA; AFE, PE, **sepsis (13%), DIC (22%)**. Qualified by either LOS >99th percentile or medical transfer, or death.	1.3 (2003–6)
CDC, Callaghan 2012^35^	NIS (USA), ICD-9-CM	Review of ICD-9 codes associated with inpatient mortality; Callaghan 2008^29^; Kuklina 2009.^31^	Expansion of CDC definition (Callaghan 2008) with aortic disease, ARDS, sickle crisis, intracranial injuries, internal injuries (non-iatrogenic), procedures on heart and pericardium. Major components were **DIC (10%)** and **transfusion (75%)**. Diagnostic codes qualified by LOS >90th percentile for mode of birth (vaginal, primary CS, repeat CS).	Del 12.9 (2008–9)
CDC+, Lyndon 2012^36^	DD, vital statistics (CA, USA)	Based on Callaghan 2008^29^ and Kuklina 2009.^31^	Cardiac arrest, HF, RF, ARDS, ARF, stroke, VST, shock, CoA, death; haemorrhage with coag. defect, DVT, PE, sepsis, DIC, uterine rupture, wound complication; ventilation, **hysterectomy (13%)**, haemorrhage with transfusion or surgical management, curettage, unplanned postnatal operation, ITU. Diagnostic codes qualified by LOS >90th percentile for type of birth (diagnostic components with LOS qualifier applied are appear unreported).	Del 5.8 (2005–7)
Wahlberg 2013^37^	National registry (Sweden)	Informed by WHO definition in Say 2009.^25^	Cardiac arrest, hypertensive HF, pulmonary oedema, MI, acute ischaemic heart disease, arrhythmia, aortic disease, ARDS, RF, ARF, LD, stroke, VST; **HELLP, eclampsia, DIC (23% combined)**, AFE, PE **(15%, obstetric embolism), uterine rupture or obstetric laparotomy (40% combined)**, sepsis, **shock or hysterectomy (11%).**	2.9 (1998–2007)
Bateman 2013^38^	Medicaid healthcare utilisation dataset (USA)	Developed from Callaghan 2008,^29^ Callaghan 2012,^35^ Mhyre 2011^34^; end-organ injury based definition.	**HF (20%)**, **pulmonary oedema (10%)**, MI, **ARDS/RF (14% combined)**, SA, ARF, **LD (11%)**, stroke, VST, shock, coma, SE, delirium, death; PE, **sepsis (22%)**, **DIC (14%)**.	11.6 (2000–7)
Non-transfusion CDC, Creanga 2014^39^	SID (USA), ICD-9-CM	As per Callaghan 2012.^35^	CDC components with the omission of transfusion-only cases.	Del 6.4 (2008–10)
Lisonkova 2014^40^	State registry (WA, USA), ICD-9	SMM defined as any potentially life-threatening condition.	Cardiac arrest, HF, pulmonary oedema, MI, cardiomyopathy, malignant hypertension, arrhythmia, haemopericardium, aortic disease, pulmonary arterial aneurysm, RF, ARDS, ARF, LD, stroke, VST, shock; AFE, DVT, PE, DIC, **chorioamnionitis/infection/sepsis (71% combined)**; hysterectomy, **transfusion (17%, any product)**. Defined with and without **“obstetric trauma”** (uterine rupture or inversion, pelvis or pelvic organ injury, cervical/high vaginal laceration, third and fourth degree perineal tear; **76%, combined, SMM with trauma cases**).	26.3 (2000–08), without trauma (late-onset pre-eclampsia model)
Pallasmaa 2015^41^	National registry (Finland), ICD-10	Based on review of all diagnostic and procedural codes assigned to deliveries over a five year period.	Pulmonary oedema, ARDS, shock, peritonitis; haemorrhage with coag. defect, AFE, DVT, PE, VST, uterine rupture, uterine inversion, ileus/bowel obstruction, **sepsis (52%)**, CoA; hysterectomy, surgical management of haemorrhage, repair of wound dehiscence, **laparotomy (haemorrhage) (13%)**, laparotomy (other causes), laparoscopy.	D42 12.8 (2007–12)
Schummers 2015^42^	Perinatal registry (British Columbia, Canada)	Apparent subset of CPSS codes.	Cardiac arrest, HF, pulmonary oedema, MI, ARDS, ARF, cardiomyopathy, stroke, VST, shock, death; APH with coag. defect, PE, AFE; repair of organ injury. Components not reported individually.	6.0 (2004–12)
EMMOI, Nair 2016^16^	HES (England, UK)	Based on Roberts 2008^17^; modified according to the quality of administrative data in the UK.	Supplementation of AMMOI with diagnostic codes for eclampsia and puerperal sepsis. Excluded isolated transfusion. Major components included **uterine rupture (13%)**, **eclampsia (19%)**, **sepsis (11%)**; **evacuation of haematoma (13%)**.	4.7 (2012–13)
Main 2016^12^	DD-vital records (CA, USA), ICD-9 CM	Validation study.	Various modifications of the CDC index with the addition of either ≥4 units transfusion (any) or ≥4 units transfusion (red cells) or ITU admission or pLOS or ITU/pLOS	8.6; 8.0; 13.5; 16.4 17.0, respectively
CDC-NYS, Lazariu 2017^43^	DD-vital records (NY, USA), ICD-9 CM	Expansion of CDC criteria based on literature review.	CDC with additional ‘New York supplemental codes’ (NYS) qualified by pLOS or ITU admission. Supplementary codes for pulmonary oedema, cardiac complications, LD, ARDS, shock, anaemias, coma, DKA, delirium, SA, SE, thyroid crisis, oliguria; haemorrhage, DIC, eclampsia, sepsis, PE, DVT, VST, thrombocytopenia. Substantial components included **transfusion (22%**) and **anaemias/sickle cell (47%, combined)** based on 2013 numerator rate and 2008–13 denominator rate.	Del 25.5 (95% 25.2, 25.7), 2008–13
Abe 2018^44^	Diagnosis Procedure Combination database (Japan), ICD-10	Unspecified; disease-specific and management based selection of life-threatening conditions.	HF, MI, acute pericarditis, cardiomyopathy, aortic disease, cardiac CoA **(all cardiovascular combined 14%)**, pulmonary oedema, ARDS, RF, respiratory CoA, pneumonia, **(all respiratory combined 10%)**, ARF, stroke; PE, sepsis; **transfusion on day of delivery (red cells, 53%)**, **transfusion on day of delivery (FFP, 37%)**, shock treated with vasopressors.	Selected population
Non-transfusion CPSS, Aoyama 2019^45^	DD (Canada), ICD-10CA	As per Joseph 2010.	CPSS components with the omission of transfusion-only cases. Major components included **septicaemia during labour/puerperal sepsis (22%)**.	17.7 (2004–15)
CPSS, Dzakpasu 2020^14^	DD (Canada), ICD-10CA	From Joseph 2010. Candidate components reviewed according to incidence, length of stay, and mortality rates.	As per Joseph 2010 with addition of **severe pre-eclampsia, HELLP (35% combined, with eclampsia)**, acute fatty liver disease with transfusion, uterine inversion at vaginal birth, and **ITU (12%)** and removal of HIV, pre-existing hypertensive cardiac disease, and evacuation of haematoma without transfusion. Transfusion (red cells) included only with defined diagnostic codes. Other major components were **haemorrhage + transfusion/coag. defect, curettage + transfusion (31% combined)**, and **surgical complications/evacuation of incisional haematoma + transfusion, repair of organ injury, reclosure of CS wound + transfusion (12% combined)**.	Del 16.1 (2012–16)
Duke 2020^46^	Victorian Admitted Episode Dataset (Victoria, Australia)	Developed from WHO MNM tool^25^ and Main 2016.^12^	Cardiac arrest, HF, cardiomyopathy, RF, ARF, LD, **CoA/anaphylaxis (12%)**, Addisonian crisis, complication of transplanted organ; DIC, **severe pre-eclampsia/HELLP (25%),** eclampsia, AFE, PE, **sepsis (17%),** uterine rupture, **early pregnancy loss-related (11%)**; CPR/cardioversion, ventilation, hysterectomy, **PPH + surgical management/transfusion and coagulopathy/transfusion of coag. factors (17%)**, repair of organ injury, laparotomy, dialysis.	35.4 (95% CI 35.0, 35.8) (2001–2017)
CDC 2019-Bateman, Snowden 2021^47^	Statewide birth cohort/vital record-DD (CA, USA)	Compared agreement between multiple CDC-based and birth certificate-based measures. The use of this definition was to consider the effect of different sets of codes.	CDC index without transfusion with additional diagnostic codes from Bateman 2013.^38^ Components not reported individually.	9.7 (2007–12)
EMMOI-ITU, Masterson 2022^48^	Scottish Morbidity Record (Scotland)	Based on EMMOI^16^	EMMOI supplemented with **ITU (18%).** The other major component was **sepsis (35%)**.	10.4 (2005–18)
CDC, AIM 2022^49^	NA	Based on Callaghan 2012^35^	As per Callaghan 2012 with rolling update. Removal of invasive monitoring and transfusion.	–
Fridman 2023^50^	DD (CA, USA), ICD-10-CM	Modification of CDC^49^ to exclude indicators that are not predictable, pre-existing conditions or their precipitating factors, including diagnoses present on arrival.	CDC index with the exclusion of aneurysm, sickle crisis, AFE, transfusion and conditions present on admission. Components not reported individually.	4.4 (2016–18)
Tsamantioti 2024^51^	Birth register-DD (Sweden), ICD-10-SE	Review of CDC^35^^,^^49^ and CPSS definitions^14^ by a multidisciplinary group of experts to evaluate validity. Concurrent comparison of LOS for components. Addition of severe mental health components.	Cardiac arrest, HF, pulmonary oedema, MI, cardiomyopathy, ARDS, ARF, LD, stroke, shock, CoA, SE, coma, sickle crisis, acute psychosis, inpatient psychiatric care; haemorrhage with transfusion/coag. defect (**PPH with transfusion, 39%**), DIC, **severe pre-eclampsia/HELLP (37%, combined)**, eclampsia, AFE, PE, VST, sepsis, obstetric haematoma, suicide attempt; CPR/cardioversion, ventilation, hysterectomy, uterine rupture with transfusion or repair of rupture with transfusion, **curettage/manual exploration of uterine cavity with transfusion (20%)**, dialysis.	27.2 (95% CI 26.8, 27.2) (1999–2019)
CDC-Scotland, Kearns 2024^52^	DD-vital record (Scotland), ICD-9 and ICD-10	Application/adaptation of CDC criteria^49^ for use with Scottish administrative data.	CDC criteria with additional qualifier of critical care admission applied to postpartum haemorrhage and to sepsis due to over-estimation.	Selected population

CDC, Centres for Disease Control and Prevention; CPSS, Canadian Perinatal Surveillance System; AMMOI, Australian Maternal Morbidity Obstetric Indicator; EMMOI, English Maternal Morbidity Obstetric Indicator; WHO, World Health Organization; DD, Discharge data; NSW, New South Wales; NHDS, National Hospital Discharge Survey; NIS, National Inpatient Sample; HES, Hospital Episode Statistics; HF, heart failure; MI, myocardial infarction; RF, respiratory failure; ARDS, acute respiratory distress syndrome; SA, status asthmaticus; ARF, acute renal failure; LD, liver disease (acute); SE, status epilepticus; CoA, complications of anaesthesia; DKA, diabetic ketoacidosis; PPH, postpartum haemorrhage; APH, antepartum haemorrhage; AFE, amniotic fluid embolism; DVT, deep venous thrombosis; PE, pulmonary embolism; VTE, venous thromboembolism; DIC, disseminated intravascular coagulation; ITU, intensive treatment unit; LOS, length of stay; pLOS, prolonged length of stay.

Components of definitions which represented more than 10% of cases of SMM are noted (%) and shown in bold. Components are not mutually exclusive. Del denotes the incidence represents SMM at the delivery episode, D42 denotes delivery episode and up to 42 postnatal days.

**Table 2:** Definitions of SMM developed for use with the primary medical record.

Definition	Data	Development	Description	Incidence (/1000)
Mantel criteria, Mantel 1998^53^	Prospective case review (Pretoria region, South Africa)	Unspecified.	Cardiac arrest, **pulmonary oedema (16%)**, RF, ARF, oliguria, coma, haemorrhagic stroke, DKA, thyroid crisis, CoA; pre-eclampsia with jaundice; ventilation, **hysterectomy (29%)**, **hypovolaemia with ≥5 units whole blood/red cells (27%)**, acute thrombocytopenia with platelet transfusion, ITU	10.9 (1996)
SCASMM precursor, Brace 2004^54^	Prospective systematic case surveillance (Scotland)	Based on Mantel 1998.^53^ Definitions based on pathophysiological rather than interventional measures preferenced.	Cardiac arrest, pulmonary oedema, **ARF (10%)**, anaphylaxis, cerebrovascular disease (“including haemorrhage or thrombosis”), coma, SE, CoA; **haemorrhage transfused ≥5 units (50%)**, **eclampsia (13%),** septicaemic shock; **ITU (33%)**.	3.8 (2001–02)
Geller criteria, Geller 2004^55^	Retrospective review of selected cases.	Case review of SMM to characterise and differentiate near-miss from non-near-miss/“less severe morbidity”.	‘Five-factor scoring system’ included organ system failure (“failure of either cardiac, pulmonary, hematologic, renal, liver/gastrointestinal, or central nervous system”); extended intubation, ITU, surgical intervention, transfusion ≥4 units.	NA
SAMM, Zwart 2008^56^	Prospective systematic surveillance (‘LEMMoN study’, NL)	Literature review; agreement of National Maternal Mortality Committee.	SMM as per treating obstetrician (including potential indirect processes), eclampsia, HELLP with liver haematoma/rupture, uterine rupture, **haemorrhage with transfusion ≥4 units red cells or embolization/hysterectomy (45%)**; **ITU (33%)**	7.1 (2004–6)
WHO SMM/near-miss, Say 2009,^25^ WHO 2011^23^	NA	WHO systematic review of maternal morbidity and mortality.^3^ Organ dysfunction criteria based on the Sequential Organ Failure Assessment score. Development by an expert working group and field tests of identification criteria.	**Severe maternal complications:** severe PPH, severe pre-eclampsia, eclampsia, sepsis or severe systematic infection, ruptured uterus, severe complications of abortion **Critical interventions of intensive care unit use**: ITU, interventional radiology, laparotomy, use of blood products **Maternal vital status**: maternal death In addition to categories that indicate “near-miss”: **Clinical criteria:** acute cyanosis, gasping, respiratory rate >40 or <6/min, shock, oliguria not responding to fluids/diuretics, clotting failure, LOC ≥12 h, LOC with absence of pulse, stroke, uncontrollable fit/total paralysis, pre-eclampsia with jaundice. **Laboratory criteria:** oxygen saturation <90% for ≥60 min, PaO_2_/FiO_2_ <200 mmHg, creatinine ≥300 μmoL/l or ≥3,5 mg/dl, bilirubin>100 μmoL/l or >6.0 mg/dl, pH < 7.1, lactate >5 mmol/L, acute thrombocytopenia <50,000 platelets, LOC with glycosuria and ketonuria. **Management criteria:** continuous vasoactive drugs, hysterectomy following infection or haemorrhage, transfusion ≥5 units red cells, intubation and ventilation ≥60 min not related to anaesthesia, dialysis for ARF, CPR.	NA
Murphy 2009^57^	Prospective systematic case surveillance (Dublin, Ireland)	Based on Mantel 1998.^53^	As per Mantel 1998 with the addition of eclampsia, radiologically-proven PE, and anaphylactic shock. Major components included **hypovolaemia requiring ≥5 units red cells (47%)**, **acute thrombocytopenia + platelet transfusion (19%)**, **eclampsia (15%)**, and **ITU (13%)**.	3.2 (2004–05)
SMMG, NPEC 2013^58^	Primary record (Ireland)	From the Severe Maternal Morbidity Report 2011 (NPEC 2013, earliest instance). Review of literature; based on SCASMM.	Cardiac arrest, pulmonary oedema, acute respiratory dysfunction, ARF, LD, stroke, CoA, coma, SE; eclampsia, VST, septicaemic shock; **haemorrhage ≥2500 ml or with ≥5 units of blood (53% combined)**, hysterectomy, interventional radiology, **ICU (40%)**.	6.3 (2021)
SCASMM, Marr 2014^59^	Prospective systematic case surveillance	Based on Mantel 1998^53^ and Brace 2004.^54^	Cardiac arrest, pulmonary oedema, ARF, ALD, stroke, coma, SE, CoA, anaphylaxis; eclampsia, massive PE, VST, septicaemic shock; ventilation, **haemorrhage ≥ 2500 ml or ≥5 units red cells or treated coagulopathy (67%)**, **ITU (21%)**.	6.1 (2003–12)
ACOG, Main 2016^12^	Routine screen then case review (CA, USA)	Screening criteria of a CDC code, pLOS, ITU, or transfusion. Case review by expert panel which developed criteria from Geller 2002^4^ and Geller 2004^55^ using a modified Delphi process.	Example-based guidance, including rule-out criteria (omitted here). Summary of main criteria: **Cardiac** Pre-existing cardiac disease + ITU for treatment, peripartum cardiomyopathy, arrhythmia + >1 dose antiarrhythmic or ITU, ITU admission for treatment **Respiratory** ARDS, pulmonary oedema, postoperative pneumonia, DVT or PE; ventilation **Renal** Acute tubular necrosis or dialysis, oliguria + >1 dose diuretic, acute creatinine elevation **Haemorrhagic** Haemorrhage with either: • transfusion ≥4 units • <4 units + pulmonary oedema + >1 dose of diuretic • return to theatre (major procedure) • uterine artery embolization • 2–3 units + uterine balloon/compression suture • ITU + invasive monitoring/treatment • Unplanned hysterectomy **Hypertensive** Pre-eclampsia with severe/refractory hypertension, hepatic haematoma or severe injury + ITU, HELLP; eclampsia or status epilepticus, antihypertensive infusion, non-responsiveness or loss of vision **Infective** Septic shock requiring fluid resuscitation/vasopressors, infection + pulmonary complication **Other** Organ injury, prolonged ileus, small bowel obstruction, CoA, invasive monitoring	7.3 (2012–13)
‘EPIMOMS study’, Siddiqui 2018^60^	Prospective systematic case surveillance	National Delphi-Rand process	Cardiac arrest, pulmonary oedema, decompensation of pre-existing cardiomyopathy, troponinaemia >1 μg/L, RF, ARF, LD, stroke, TIA, coma, death; **‘severe hypertensive disorder’ including placental abruption with haematological dysfunction, severe pre-eclampsia (birth <32/40), HELLP, eclampsia (19% combined)**, acute anaemia, DIC, PE, severe acute psychiatric disorder or acute decompensation of chronic psychiatric disorder or suicide attempt; **“severe obstetrical haemorrhage”including PPH ≥1500 ml, transfusion ≥4 units red cells or surgical management, hysterectomy (65% combined)**, laparotomy, ITU	NA
‘PreCARE’ study, Linard 2018^61^	Abstraction from primary record (Paris, France)	Unspecified.	Convulsions, DKA, death; haemorrhagic shock, placental abruption, **severe pre-eclampsia (28%)**, eclampsia, DVT, PE, uterine rupture, severe sepsis, **3**rd**/4**th **degree perineal tear (22%)**; **haemorrhage with second-line medical management and transfusion ≥2 units red cells or surgical management/hysterectomy (18% combined)**, **ITU (16%)**, **surgical reintervention (14%)**.	28.4 (2010–12)
Brakewood 2024^62^	Abstraction from primary record (Rhode Island, USA)	Unspecified	Pulmonary oedema, ARF; sepsis, DIC, DVT, PE; hysterectomy, blood transfusion, ITU. Components not reported individually.	Selected population
Boulet 2024^63^	Retrospective review of primary record (Atlanta, USA)	Modification of ACOG guidance (Main 2016)^12^ with further specification of the examples from Main 2016.	As per Main 2016 with additional quantification of haemorrhage (1000 mls, accompanied by treatment as per original definition); degree of fluid resuscitation in septic shock (≥3000 mls); and addition of sickle crisis and DIC. Major components included **hypertensive disease (69%)**, **haemorrhage (20%)**, **pulmonary complications (18%)**, and **renal disease (10%)**.	Selected population; single centre, high risk population, Del 74 (2016–18)

SCASMM, Scottish Confidential Audit of Severe Maternal Morbidity; WHO, World Health Organization; NPEC, National Perinatal Epidemiology Centre; CA, California; RF, respiratory failure; ARF, acute renal failure; LD, liver disease (acute); SE, status epilepticus; LOC, loss of consciousness; DKA, diabetic ketoacidosis; CoA, complications of anaesthesia; PE, pulmonary embolism; VST, venous sinus thrombosis; DIC, disseminated intravascular coagulation; CPR, cardiopulmonary resuscitation; ITU, intensive treatment unit (or similar).

Major components of definitions which represented more than 10% of cases of SMM are noted (%) and shown in bold. Components are not mutually exclusive. Del denotes the incidence represents SMM at the delivery episode, D42 denotes delivery episode and up to 42 postnatal days.

**Fig. 2:**
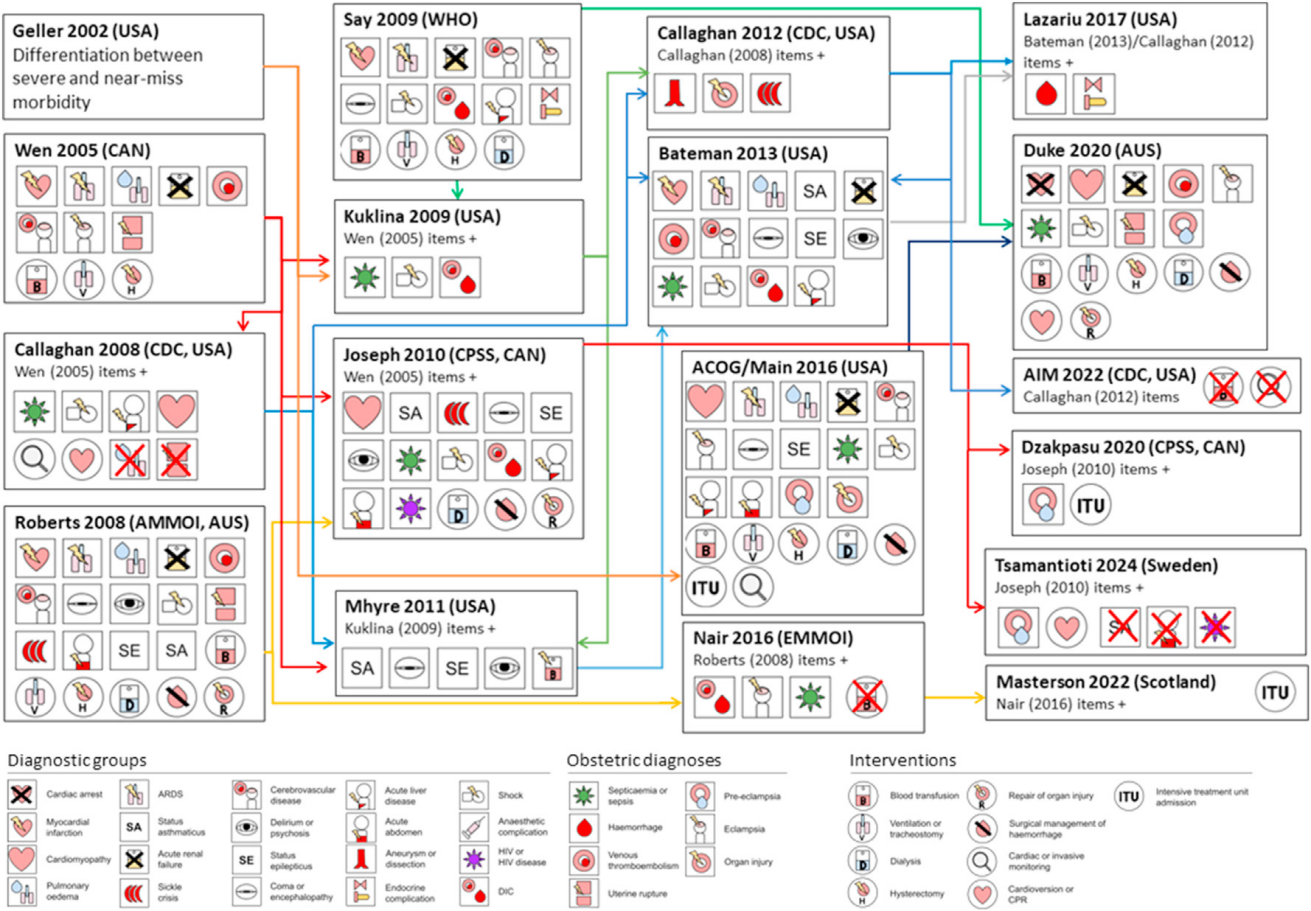
**Definitions of SMM over time**. Select, related definitions of SMM in different settings over time (left to right). An arrow denotes that a study reported its definition was informed by the earlier study. The main components of definitions are shown, including general diagnostic groups, obstetric diagnoses (both square icons), and interventions (round icons). Components added to (‘+’) or removed from (red ‘X’) previous definitions are shown. ACOG, American Congress of Obstetrics and Gynaecology; WHO, World Health Organisation maternal near-miss; CDC, Centers for Disease Control and Prevention; CPSS, Canadian Perinatal Surveillance System; AMMOI, Australian Maternal Morbidity Obstetric Indicator; EMMOI, English Maternal Morbidity Obstetric Indicator.

Of the 28 studies of definitions for use with administrative data, 13 were from the USA^12^^,^^29^^,^^31^^,^^34^, ^35^, ^36^^,^^38^, ^39^, ^40^^,^^43^^,^^47^^,^^49^^,^^50^; 5 from Canada^14^^,^^27^^,^^33^^,^^42^^,^^45^; 3 from Australia^17^^,^^28^^,^^46^; 2 from Scotland,^48^^,^^52^ 2 from Sweden^37^^,^^51^ and one each from England,^16^ Finland,^41^ and Japan.^44^ Of these, 2 studies (2/28, 7%) developed or reported definitions with reference to the primary medical record^12^^,^^17^ and 1 study cited previous case review evidence^29^; 4 studies informed the development of a definition by comparing the case fatality rates of candidate components (4/28, 14%)^27^^,^^29^^,^^34^^,^^35^; 3 informed the development of definitions by comparing the length of stay for candidate components (3/28, 11%)^14^^,^^29^^,^^51^; 3 studies developed definitions informed by the WHO definition of SMM (3/28, 11%)^31^^,^^37^^,^^46^; 3 studies developed definitions from a literature review (3/28, 11%)^17^^,^^34^^,^^43^; 12 modified or were based on previous definitions other than the WHO definition (15/28, 54%).^12^^,^^14^^,^^16^^,^^29^^,^^33^, ^34^, ^35^, ^36^^,^^38^^,^^39^^,^^45^^,^^48^, ^49^, ^50^, ^64^, ^51^ Two validation studies reported definitions as part of a theoretical comparative process without application of the definitions (2/28, 7%).^12^^,^^47^ Additionally, 4 studies compared components or SMM overall according to length of stay, prolonged length of stay, mortality, or ITU admission following development of the definition (4/28, 14%).^33^^,^^45^^,^^46^^,^^48^ The incidence of SMM according to these original studies ranged from 1.3 per 1,000^34^ to 35.4 per 1,000^46^ with a median incidence of 11.4 per 1000. Of the 13 studies of definitions for use with the primary medical record, there was the WHO definition^25^; 4 studies from the USA^12^^,^^55^^,^^62^^,^^63^; 2 from Scotland^54^^,^^59^; 2 from France^60^^,^^61^; 2 from Ireland,^57^^,^^58^ and one each from the Netherlands,^56^ and South Africa.^53^ Of these, 3 studies (3/13, 23%) developed definitions with direct reference to the primary record^12^^,^^25^^,^^55^; 3 involved a consensus process (3/13, 23%)^12^^,^^56^^,^^60^; and 5 modified or were based on previous definitions (5/13, 38%).^54^^,^^57^, ^58^, ^59^^,^^63^ The incidence of SMM according to these original studies ranged from 3.8 per 1000^54^ to 28.4 per 1000^63^ with a median incidence of 6.7 per 1000. The relationships between select definitions for administrative data and comparison of the components is shown in Fig. 2.

### Components of definitions

**Fig. 3:**
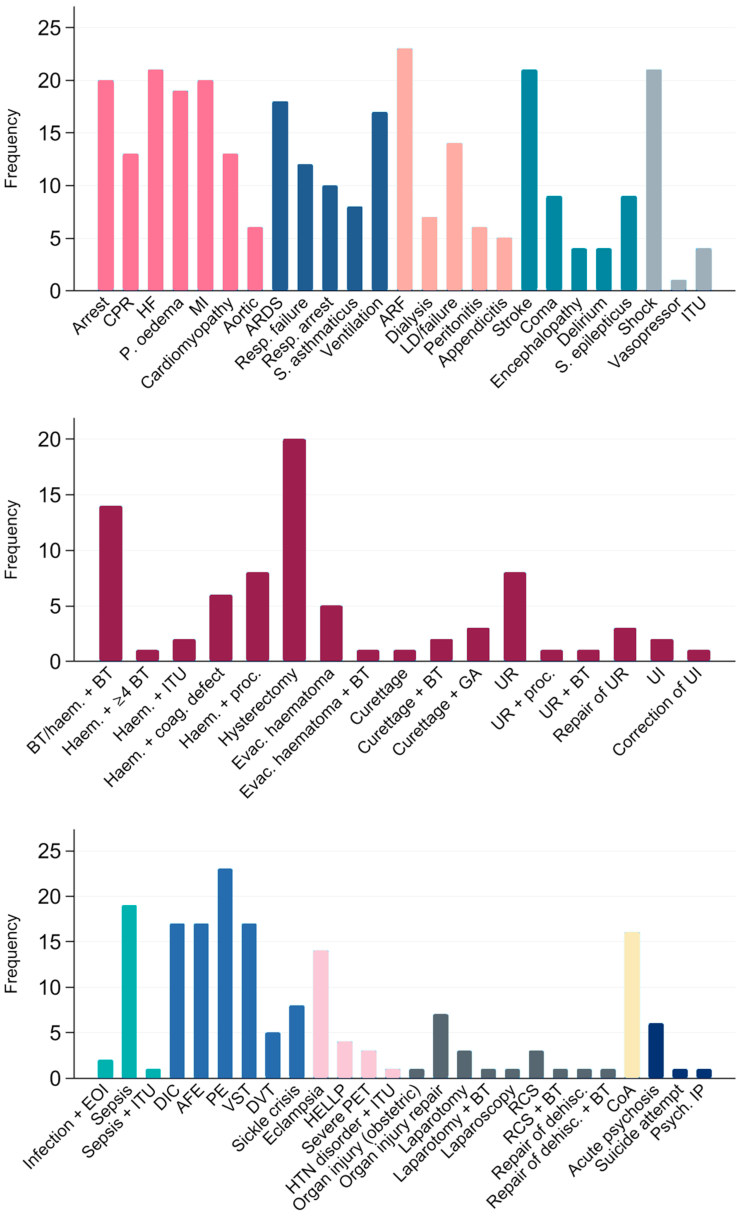
**Frequency of components within SMM indicators for use with administrative data**. CPR, cardiopulmonary resuscitation; HF, heart failure; P., pulmonary; MI, myocardial infarction; ARDS, Acute respiratory distress syndrome; Resp., respiratory; S., status; ARF, acute renal failure; LD, liver disease; ITU, intensive treatment unit; BT, blood transfusion; Haem., Haemorrhage; proc., Procedure; evac., Evacuation; GA, general anaesthetic; UR, uterine rupture; UI, uterine inversion; EOI, end organ injury; DIC, dissemination intravascular coagulation; AFE, amniotic fluid embolism; PE, pulmonary embolism; VST, venous sinus thrombosis; DVT, deep venous thrombosis; PET, pre-eclampsia; HTN, hypertensive; RCS, reclosure of caesarean section; dehisc., Dehiscence; psych., psychiatric; IP, inpatient.

Among 24/32 definitions for use with administrative data, the most common components were acute renal failure (ARF), pulmonary embolism (PE; 23/24 definitions); shock, sepsis/severe infection (22/24); heart failure (HF), stroke (21/24); cardiac arrest, myocardial infarction (MI; 20/24), pulmonary oedema (19/24), and acute respiratory distress syndrome (ARDS; 18/24). The commonest interventions were hysterectomy (20/24), ventilation (17/24), and transfusion (15/24). Conversely, examples of components included in only one SMM definition included acute fatty liver,^14^ transplant complication,^46^ panhypopituitarism,^34^ acute ischaemic cardiac disease,^37^ and Addisonian crisis.^46^ The frequency with which components were included is shown in Fig. 3. Among 11/13 studies that included definitions for use with the primary record, the most common components were ARF (10/11) eclampsia, pulmonary oedema, cardiac arrest, stroke, coma (8/11); severe sepsis/septicaemic shock, uncontrollable fit/status epilepticus (7/11) and acute liver disease, or severe pre-eclampsia/pre-eclampsia with complications (6/11). The commonest interventions were ITU admission (11/11); a transfusion of ≥4/≥5 units of blood products (9/11), hysterectomy (8/11), and ventilation (7/11). Components found in a single definition included symptom-based (e.g., gasping), vital-signs based (e.g., respiratory rate >40 or <6/minute) and laboratory-based (e.g., PaO_2_/FiO_2_ <200 mmHg, pH <7.1, lactate >5 mmol/L) components of the WHO definition^25^ although these may map to similar diagnostic components elsewhere.

There were five approaches to incorporating transfusion/haemorrhage into definitions using administrative data, including ‘any transfusion’, ‘diagnosis of haemorrhage with transfusion’, ‘procedure with transfusion’, ‘transfusion with specified blood products’, and ‘transfusion ≥4 units’, including combinations of approaches. Alternatively, definitions incorporated neither a diagnosis of haemorrhage nor transfusion and recognised only surgical management of haemorrhage, end-organ complications, or critical interventions. Definitions for use with the primary medical record were more nuanced with combinations of transfusion at different thresholds (≥2 units, ≥4 units, ≥5 units), in conjunction with thresholds of estimated blood loss (≥1500 mls, ≥2500 mls), or management criteria (second-line medical, surgical or radiological management, including hysterectomy). In most definitions for administrative data that included ‘any transfusion’/haemorrhage with ‘any transfusion’, this was the most common condition among cases (9/12, 75%). For the remaining definitions that did not include ‘any transfusion’ and reported on individual components, common conditions (assigned to >10% of cases) included sepsis (7/8, 75%), dissemination intravascular coagulation (DIC)/coagulopathy (3/8, 38%), and LD (2/8, 25%). Sepsis was the most common condition in the majority of these definitions (4/8, 50%).

### Diagnostic and procedural codes for example definitions from different countries using routine data

Diagnostic codes for the majority of components were defined similarly. The CDC (Centers for Disease Control and Prevention) definition excluded first trimester diagnoses, where this level of definition was available. The codes for pulmonary oedema, HF, cardiomyopathy, ARDS, coma, status epilepticus (SE), appendicitis, psychosis, amniotic fluid embolism (AFE), uterine rupture, and DIC were near-identical. Codes for respiratory conditions demonstrated variation. The CDC definition included codes for respiratory distress, failure, and arrest as well as procedure-related insufficiency whereas other definitions focussed on ARDS or else captured respiratory failure through ventilation only. Separate codes for procedure-related diagnoses also led to variation for cardiac arrest, ARF, cerebrovascular disease, and sepsis. Complications of anaesthesia (CoA) are represented across several ICD categories for pregnancy, labour and delivery, or the puerperium and whilst most codes were consistent, there was variation according to the time periods included. The CDC definition included additional codes for transient ischaemic attack (TIA), vascular syndromes, and encephalopathies, and the CDC and CPSS (Canadian Perinatal Surveillance System) included additional codes for MI. Haemorrhage was defined using diagnostic codes for antepartum haemorrhage (APH)/intrapartum haemorrhage (IPH) with clotting abnormality (CDC, CPSS, Finnish), codes for haemorrhage without clotting abnormality qualified by additional procedural/transfusion codes (CPSS), any transfusion (CDC, AMMOI), hysterectomy (all), surgical or radiological management (AMMOI, EMMOI, CPSS, Finnish), or balloon tamponade (CPSS, Finnish), specific complications that may relate to haemorrhage such as DIC (CDC, AMMOI, EMMOI, CPSS, Swedish), or through end-organ complications or shock (all). For hypertensive disease, most included eclampsia (CDC, EMMOI, CPSS, Swedish), fewer included HELLP syndrome (CPSS, Swedish), or severe pre-eclampsia (CPSS), or no diagnoses, instead relying on end-organ complications and critical interventions (AMMOI). Puerperal sepsis codes were included in most definitions and additional severe sepsis and septic shock codes were included in the CDC definition whereas the CPSS definition included “septicaemia during labour”. Further additions included sepsis due to specified organisms, included in the CDC (streptococcal, listerial, other), and Wahlberg (streptococcal, listerial, actinomycosis, candidal) definitions. Codes included in example definitions across example definitions are shown in Supplementary Table S3.

## Discussion

There were 32 composite definitions for administrative data and 13 for use with the primary medical record. Only a minority of definitions for administrative data were directly informed by reviews of cases of SMM or compared with other measures of severity. There were over 50 broad clinical concepts among the constituents of these comprehensive definitions. The majority of definitions sought to capture severe cardiac, pulmonary, and renal morbidity; specific clinical severe morbidities including cerebrovascular, thromboembolic, haemorrhagic and infective disease, as well as shock; urgent obstetric interventions such as hysterectomy; and critical interventions such as ventilation. The pattern of inclusion for other direct disease processes and a wide range of indirect disease processes varied. The majority of studies that developed definitions for administrative data considered only the delivery admission.

There was broader agreement for the inclusion of diagnostic codes reflective of end-organ injury and critical interventions. The approach to other diagnostic codes differed, particularly related to direct disease processes where codes are not necessarily as informative of disease severity. For example, the ICD code O75.3 is varyingly interpreted to represent sepsis in labour or septicaemia in labour or other infections in labour - a broad spectrum. Only a minority of studies informed the development of definitions with or validated definitions of SMM at the time of development according to length of stay or case fatality rate which, compared with unexposed women, guides the interpretation of individual codes and definitions. The reliability of administrative data is dependent on the construction of an accurate clinical narrative, its correct interpretation and assignment of codes.^64^ Several of the commonest components have been identified to have poor positive predictive values in validation against the primary medical record, including ARF and cerebrovascular disorders,^65^^,^^66^ although these may vary between settings. Some diagnostic codes relate to specific diseases but these have a broad spectrum of potential severity, such as pre-eclampsia.^67^ Procedures may be easier to capture using administrative data, compared with diagnoses or organ injury^68^^,^^69^ but require a clear indication.

There was variation in the proportion of cases identified by components within definitions; for example, the proportion of women who had haemorrhage with transfusion varied between 21% and 75% of women with SMM within administrative definitions. Whilst definitions may be reported or interpreted as measuring severe morbidity, they may reflect and track one dominant disease process. Although a serious decision, transfusion may also represent non-severe or non-acute or pre-existing morbidity. The positive predictive value of any-transfusion for clinically-validated SMM has been found to vary from moderate^47^ to poor^47^^,^^65^^,^^66^ but its use has persisted, alongside reporting of SMM without transfusion. One reason may be that in the absence of additional blood bank data, ‘any transfusion’ can increase the sensitivity of an indicator.^63^ Nevertheless, the positive predictive value of the CDC definition for clinically-validated SMM may be higher without transfusion.^12^^,^^66^ Composite measures should be carefully evaluated before use for prediction or for evaluation of interventions so that these processes are oriented towards SMM and not commoner but less important components.^70^ An alternative approach could be to weight components according to their importance; for example, using a hierarchical composite endpoint.^71^ Transfusion rates can inform quality improvement separately when it is not possible to identify the number and type of blood product administered.^12^

Relatedly, different components within individual definitions potentially capture different underlying healthcare processes. Cardiac arrest and severe perineal tear represent different dimensions of morbidity, pose a different risk of progression to death, and would involve different quality improvement processes. Additionally, definitions were often informed by the concepts of end-organ injury and acuity of treatment but not the views of patients or the public. The subjective and experiential aspects of SMM^72^ are important but difficult to measure and integrate into definitions; nevertheless, patient and public involvement in the development of definitions should be considered.

The principles that composite measures should consist of valid,^29^ unidimensional constituent components^73^ of a comparable incidence and importance^74^ are applicable to SMM. Several studies also provided information on sub-groups of SMM, which could support the understanding of what aspects of SMM potential interventions act on. Of studies that reported individual groups, in all cases these were based on either ICD code categories or individual organ systems. The report of subgroups according to ICD code categories or organ systems is less informative because these groups contain diseases with different determinants and processes. For example, “cerebrovascular disorders” includes ischaemic and haemorrhagic strokes, venous sinus thromboses, and various encephalopathies. Other proposed subgroups involve similar organ-system based classification^75^ but a lack of granularity may lead to misclassification of disease processes at the code level.^75^ The organ systems approach could be adapted to a disease process approach with the correct assignment of complications such as ARF, DIC, HF, or shock to the causative process. Another approach has been to report subgroups according to the WHO categories of severe complications, critical interventions, and organ dysfunction.^76^ It would be most informative to be able to identify disease processes that can be acted on, and within those the fraction critically unwell. The principles of composite measures are important for applications where SMM may not be disaggregated into subgroups, such as clinical prediction. Whatever the application, it is important for researchers to consider whether their selected definition is suitable; reporting why a definition was selected would assist readers to judge whether it aligns with the stated objectives.

The components of composite measures and the time over which they are measured should be guided by the purpose of the measure. For example, indicators with and without transfusion may provide different insight into the effect of improvement interventions.^77^ If modifiability of the SMM indicator is desirable, components such as pre-existing disease, disease pathways that cannot be modified within the timeframe of maternity care, and disease processes with a natural course that does not permit timely intervention may be undesirable. We identified one definition relating to ‘preventable SMM’^50^ that excluded morbidity present on admission (birth admission), focussing its application to the immediate complications of labour and birth. There were no definitions related to a ‘preventable’, or modifiable, SMM from a maternity system perspective. In fact, most definitions based on administrative data were applied to the birth admission only whereas approximately 30% of SMM may occur either before or after this period.^46^^,^^56^^,^^78^ A range of severe non-obstetric conditions occur predominantly before labour and birth^48^; equally, some complications related to birth may occur predominantly on postnatal readmission.^45^ Delivery admission indicators may be appropriate for peripartum interventions^77^ but distal exposures require a broader time horizon.^45^ It is not only important to measure this morbidity but to recognise that the care provided by the maternity system outside of the birth admission can also be optimised to target reductions in antenatal and postnatal SMM.

International comparison of practice and policy can identify opportunities to improve maternity care, particularly where there is insufficient variation in clinical practice for comparison in a single country.^13^^,^^79^ We did not identify any internationally-developed definitions of SMM for use with administrative data. The WHO definition itself cannot be directly applied to administrative data and the application of its diverse criteria to administrative data may cause variation.^37^ Additionally, there were key differences between definitions developed in different countries that limit comparability. These included recognising different thresholds for severe disease within disease pathways, inclusion of different sets of diagnoses, and inclusion of process measures such as ITU admission. Definitions for the comparison of healthcare systems should be informed by knowledge of clinical practice and resource allocation given that the availability and threshold for intervention may differ,^17^^,^^80^ complicating comparisons aside from the availability of data.^16^ The ideal composite measure would therefore exclude measures that do not represent outcomes or whose meaning may differ contextually,^81^ particularly those that identify a large proportion of cases.^76^ An internationally-comparable subgroup of conditions would be easy to implement, once agreed.^82^ Recent studies have involved the reinterpretation of definitions developed in one setting for new international applications,^51^^,^^52^ typically requiring some modification based on coding or clinical practice. The alignment of end-organ injury and critical interventions into a core group of conditions would be straightforward. Consensus processes could aim to establish what other diagnoses should be included and how these definitions might be standardised, particularly relating to the common direct disease processes.

This research was limited to mapping and comparing the contents of composite definitions only. We did not compare the use of definitions or their validation beyond their initial development. This means that a definition could be included even it was used only once, whereas definitions such as the CDC definition have been widely adopted. We sought to describe and contrast definitions in an informative but useable way; we recommend review of the individual lines of ICD code in Appendix 1 to understand examples of further nuances. Although we have described variation between diagnoses and potential reasons where evident, there may be other reasons related to context-specific clinical practice or clinical coding practice; however, we have also described definitions for use with the primary medical record, enabling comparison with components when unrestricted by administrative data.

Routine data can be leveraged to provide insight into SMM and improve the quality of maternity care but current definitions are not comparable between settings. Composite measures of SMM vary, as do the incidence and severity of individual components, which complicates their interpretation. In order to make international comparisons at scale, consensus definitions should be developed based on defined, validated, components from universally available administrative data. The purpose of definitions could be considered further, recognising that the ideal components vary by the intended application.

## Contributors

IH, SG, PW, MK conceived of and designed the study. IH conducted the literature search. IH and RL screened the literature and collected the data. IH and RL accessed and verified the underlying data. All authors contributed to interpretation of the findings. IH drafted the manuscript. RL, PW, MK reviewed and edited the manuscript. SG, JMcL, PW, MK supervised the study.

## Data sharing statement

The findings are based on a synthesis of published studies. Should any additional information regarding the data extracted for this work be required, please contact the corresponding author.

## Declaration of interests

PW is in receipt of institutional grants from the National Institute for Health and Care Research. MK is in receipt of institutional grants from the National Institute for Health and Care Research and the Healthcare Quality Improvement Partnership.

## Appendix A Supplementary data

Supplementary Table S1 Search Strategy.

Supplementary Table S2 PRISMA Checklist for the Reporting of Systematic Reviews.

Supplementary Table S3 ICD-10 Code Comparisons for Select Definitions.
