# Supplementary material for: Severe maternal morbidity in the high income setting: a systematic review of composite definitions
Source: eClinicalMedicine. 2025 Feb 13;81:103105. doi: 10.1016/j.eclinm.2025.103105 (PMC11874727; doi:10.1016/j.eclinm.2025.103105)
Supplement: Supplementary Table S1 — Search Strategy. [file mmc1.docx]

**Search Strategy**

| **Search** | **Query** | **Records** |
| --- | --- | --- |
| 9 | 7 AND 8 | 6,800 |
| 8 | "risk factor"[Title/Abstract] OR "determinant"[Title/Abstract] OR “predict*”[Title/Abstract] OR “surveillance” [Title/Abstract] OR “monitor*”[Title/Abstract] | 3,465,163 |
| 7 | 1 OR 4 OR 6 | 32,152 |
| 6 | 5 + 3 | 12,734 |
| 5 | "intensive care"[Title/Abstract] OR "ICU"[Title/Abstract] OR "intensive treatment"[Title/Abstract] OR "ITU"[Title/Abstract] OR “cardiac arrest” [Title/Abstract] | 273,415 |
| 4 | 2 + 3 | 118 |
| 3 | “maternal”[Title/Abstract] OR “pregnan*”[Title/Abstract] OR “obstetric*”[Title/Abstract] | 676,536 |
| 2 | "potentially life threatening complication"[Title/Abstract] OR “PLTC”[Title/Abstract] | 1,851 |
| 1 | "maternal morbidity"[Title/Abstract] OR “maternal near miss”[Title/Abstract] OR "maternal mortality"[Title/Abstract] OR "maternal mortality"[MeSH Terms] | 20,773 |
