# Supplementary material for: Severe maternal morbidity in the high income setting: a systematic review of composite definitions
Source: eClinicalMedicine. 2025 Feb 13;81:103105. doi: 10.1016/j.eclinm.2025.103105 (PMC11874727; doi:10.1016/j.eclinm.2025.103105)
Supplement: Supplementary Table S3 — ICD-10 Code Comparisons for Select Definitions. [file mmc3.docx]

**Comparison of Select SMM ICD-10 Codes­­**

|  |  | **CDC (AIM), USA  ICD-10** | **AMMOI (Roberts), Australia ICD-10-AM** | **EMMOI (Nair), England ICD-10** | **CPSS (Dzakpasu), Canada ICD10-CA** | **Pallasmaa, Finland ICD-10** | **Wahlberg, Sweden ICD-10** |
| --- | --- | --- | --- | --- | --- | --- | --- |
|  |  |  |  |  |  |  |  |
| **DIAGNOSTIC, OBSTETRIC** |  |  |  |  |  |  |  |
|  |  |  |  |  |  |  |  |
| **Haemorrhage** |  |  |  |  |  |  |  |
| APH, clotting abnormality |  | O46.002-.003,  O46.009, O46.012-.013, O46.019, O46.022-.023,  O46.029, O46.092-.093, O46.099 |  |  | O46.0 |  |  |
| IPH, clotting abnormality |  | O67.0 |  |  | O67.0 | O67.0 |  |
| IPH, transfusion |  |  |  |  | O67 (+transfusion) |  |  |
| Placental abruption, clotting abnormality |  | O45.002-.003,  O45.009, O45.012-.013, O45.019,  O45.022-.023, O45.029,  O45.092-.093 O45.099 |  |  |  |  |  |
| Placental abruption, clotting  abnormality + transfusion |  |  |  |  | O45.0 (+transfusion) |  |  |
| Placenta praevia + transfusion |  |  |  |  | O44.1 (+transfusion) |  |  |
| PPH + surgical management |  |  |  |  | O72 (+procedural code) |  |  |
|  |  |  |  |  |  |  |  |
| **Hypertensive disease** |  |  |  |  |  |  |  |
| Eclampsia |  | O15 |  | O15 | O15 |  | O15 |
| HELLP syndrome |  |  |  |  | O14.2 |  | O14.2 |
| Severe pre-eclampsia |  |  |  |  | O14.1 |  |  |
|  |  |  |  |  |  |  |  |
| ***Other*** |  |  |  |  |  |  |  |
| Acute fatty liver |  |  |  |  | O26.6 + transfusion |  |  |
| Complication of obstetric procedure, other |  |  |  |  |  | 669.4 |  |
| Uterine inversion |  |  |  |  |  | O71.2 |  |
|  |  |  |  |  |  |  |  |
| ***Amniotic fluid embolism*** |  |  |  |  |  |  |  |
| AFE, 1st trimester |  |  | O88.1 | O88.1 | O88.1 | O88.1 | O88.1 |
| AFE, 2nd/3rd/unspecified trimester,  childbirth, puerperium |  | O88.112-113, O88.119, |  |  |  |  |  |
|  |  |  |  |  |  |  |  |
| ***Uterine rupture*** |  |  |  |  |  |  |  |
| Uterine rupture |  |  | O71.0, O71.1 | O71.0, O71.1 |  | O71.0, O71.1 | O71.0, O71.1 |
| Uterine rupture + transfusion  or surgical management |  |  |  |  | O71.0, O71.1 (+procedural code) |  |  |
|  |  |  |  |  |  |  |  |
| **DIAGNOSTIC, NON-SPECIFIC** |  |  |  |  |  |  |  |
|  |  |  |  |  |  |  |  |
| ***Abdominal*** |  |  |  |  |  |  |  |
| Acute abdomen |  |  |  |  |  |  |  |
| Acute or unspecified appendicitis |  |  | K35, K37 | K35, K37 | K35, K37 |  |  |
|  |  |  |  |  |  |  |  |
| Peritonitis (generalised, unspecified, pelvic) |  |  | K65.0, K65.9,  N73.3, N73.5 | K65.0, K65.9,  N73.3, N73.5 | K65, N73.3, N73.5 | K65.0, K65.9 |  |
| Peritonitis (peritoneal abscess, spontaneous bacterial, choleperitonitis, sclerosing mesenteritis, other) |  |  |  |  |  |  |  |
| Paralytic ileus and instestinal obstruction  without hernia |  |  |  |  |  | K56 |  |
|  |  |  |  |  |  |  |  |
| ***Anaesthetic*** |  |  |  |  |  |  |  |
| Pulmonary complications, pregnancy |  |  | O29.0 |  | O29.0 |  |  |
| Other cardiac complications,1st trimester |  |  | O29.1 | O29.1 | O29.1 |  |  |
| Other cardiac complications,  2nd/3rd/unspecified trimester |  | O29.192, O29.193,  O29.199 |  |  |  |  |  |
| Cerebral anoxia due to anaesthesia,  1st trimester |  |  | O29.2 | O29.2 | O29.2 |  |  |
| Cerebral anoxia due to anaesthesia, 2nd/3rd/unspecified trimester |  | O29.212, O29.213,  O29.219 |  |  |  |  |  |
| Other CNS complications,  1st trimester |  |  |  |  |  |  |  |
| Other CNS complications,  2nd/3rd/unspecified trimester |  | O29.292, O29.293,  O29.299 |  |  |  |  |  |
| Aspiration pneumonitis due to anaesthesia,  labour and delivery |  | O74.0 | O74.0 | O74.0 | O74.0 | O74.0 |  |
| Cardiac complication (arrest or failure) due to anaesthesia, labour/delivery |  | O74.2 | O74.2 | O74.2 | O74.2 |  |  |
| CNS complication (anoxia) due to anaesthesia, labour/delivery |  | O74.3 | O74.3 | O74.3 | O74.3 |  |  |
| Other pulmonary complication  due to anaesthesia, labour/delivery |  | O74.1 |  | O74.1 | O74.1 |  |  |
| Pulmonary/cardiac/CNS complications  of anaesthesia, puerperium |  | O89.0, O89.1, O89.2 | O89.0, O89.1, O89.2 | O89.0, O89.1, O89.2 | O89.0, O89.1, O89.2 |  |  |
| Malignant hyperthermia |  | T88.3XXA |  |  |  |  |  |
|  |  |  |  |  |  |  |  |
| ***Cardiovascular*** |  |  |  |  |  |  |  |
| Acute HF/pulmonary oedema |  |  |  |  |  |  |  |
| Acute pulmonary oedema |  | J81.0 | J81 | J81 | J81 | J81 | J81 |
| Left ventricular failure |  | I50.1 | I50 | I50 | I50 |  |  |
| Systolic HF |  | I50.21, I50.23, [I50.20] |  |  |  |  |  |
| Diastolic HF |  | I50.31, I50.33, [I50.30] |  |  |  |  |  |
| Combined HF |  | I50.41, I50,43, [I50.40] |  |  |  |  |  |
| Right heart failure/cor pulmonale |  | I50.810, I50.811,  I50.813, I50.814 |  |  |  |  |  |
| Heart failure (biventricular, high output, end stage) |  | I50.82, I50.83, I50.84 |  |  |  |  |  |
| Heart failure (other/unspecified) |  | I50.89, I50.9 |  |  |  |  |  |
| Post-procedure heart failure |  | I97.13 |  |  |  |  |  |
| Heart failure due to anaesthesia, 1st trimester |  |  | O29.1 | O29.1 | O29.1 |  |  |
| Heart failure due to anaesthesia,  2nd-3rd/unspecified trimester |  | O29.122, O29.123,  O29.129 |  |  |  |  |  |
| Hypertensive disease (+/- CKD +/- ESRF)  with heart failure |  |  |  |  |  |  | I11.0 |
|  |  |  |  |  |  |  |  |
| Aortic aneurysm/dissection (intact/ruptured) |  | I71 |  |  |  |  | I71.0, 171.1, 171.5,  171.8 |
| Aneurysm of aorta in other diseases |  | I79.0 |  |  |  |  |  |
|  |  |  |  |  |  |  |  |
| **Cardiac arrest** |  |  |  |  |  |  |  |
| Cardiac arrest/ventricular fibrillation |  | I46 | I46 | I46 | I46 |  | I46 |
| Cardiac arrest due to anaesthesia,  1st trimester |  |  | O29.1 | O29.1 | O29.1 |  |  |
| Cardiac arrest due to anaesthesia,  2nd-3rd/unspecified trimester |  | O29.112, O29.113,  O29.119 |  |  |  |  |  |
| Cardiac arrest, post-procedure or  intraoperative |  | I97.12, I97.711 |  |  | O75.4 (inclusive of arrest) |  |  |
|  |  |  |  |  |  |  |  |
| Ventricular fibrillation and flutter |  | I48.0 |  |  |  |  |  |
| Other cardiac arrhythmias |  |  |  |  | I49 |  | I49.0 (ventricular) |
|  |  |  |  |  |  |  |  |
| **Cardiomyopathy** |  |  |  |  |  |  |  |
| Peripartum cardiomyopathy |  |  | O90.3 | O90.3 | O90.3 |  |  |
| Cardiomyopathy |  |  | I42, I43 | I42, I43 | I42, I43 |  |  |
|  |  |  |  |  |  |  |  |
| **Myocardial infarction** |  |  |  |  |  |  |  |
| Myocardial infarction, acute |  | I21 | I21 | I21 | I21 |  | I21 |
| Myocardial infarction with subsequent STEMI/NSTEMI |  | I22 |  |  | I22 |  |  |
| Ischaemic heart disease,  other acute/unspecified |  |  |  |  |  |  | I24.8, I24.9 |
|  |  |  |  |  |  |  |  |
| **Respiratory** |  |  |  |  |  |  |  |
| ARDS |  | J80 |  |  | J80 | J80 | J80 |
| Acute respiratory distress/respiratory arrest |  | R06.03, R09.2 |  |  |  |  | R09.2 |
| Acute pulmonary insufficiency/failure, post procedure |  | J951, J95.2, J95.82 |  |  |  |  |  |
| Chronic pulmonary insufficiency  following surgery |  | J95.3 |  |  |  |  |  |
| Respiratory failure (acute, acute and chronic) |  | J96.0, J96.2 |  |  |  |  | J96.0 |
| Respiratory failure (acuity unspecified) |  | J96.9 |  |  |  |  |  |
|  |  |  |  |  |  |  |  |
| Status asthmaticus |  |  |  |  |  |  |  |
|  |  |  | J46 | J46 | J45.01, J45.11, J45.81, J45.91 |  |  |
|  |  |  |  |  |  |  |  |
| **CNS** |  |  |  |  |  |  |  |
| ***Coma*** |  |  |  |  |  |  |  |
| Coma, persistent vegetative state |  |  | R40.2 | R40.2 | R40.2 |  |  |
|  |  |  |  |  |  |  |  |
| Cerebral oedema |  |  | G93.6 | G93.6 | G93.6 |  |  |
|  |  |  |  |  |  |  |  |
| ***Cerebrovascular disorders (puerperal)*** |  |  |  |  |  |  |  |
|  |  |  |  |  |  |  |  |
| *Ischaemic* |  |  |  |  |  |  |  |
| Anoxic brain damage |  |  |  |  |  |  | G93 |
| Cerebral infarction |  | I63 | I63 | I63 | I63 |  | I63 |
| Cerebral infarction, DVT in cerebral vein |  |  |  |  |  | I63.6 |  |
| Cerebral venous thrombosis,  1st trimester |  |  |  |  | O22.51 |  | O22.5 |
| Cerebral venous thrombosis/thrombophlebitis,  2nd trimester-puerperium or unspecified |  | O22.50, O22.52, O22.53 |  |  | O22.5 |  |  |
| Cerebral venous thrombosis, puerperium |  | O87.3 |  | O87.3 | O87.3 | O87.1 | O87.3 |
| Cerebrovascular infarction (perioperative) |  | I97.81, I97.82 |  |  |  |  |  |
| TIA/related syndromes,  transient retinal artery occlusion |  | G45, H34.0 |  |  |  |  |  |
| Vascular syndromes of brain |  | G46 |  |  |  |  |  |
|  |  |  |  |  |  |  |  |
| *Haemorrhagic* |  |  |  |  |  |  |  |
| Haemorrhage, atraumatic (subarachnoid, intracerebral, other/unspecified) |  | I60, I61, I62 | I60, I61, I62 | I60, I61, I62 | I60, I61, I62 |  | I60, I61, I62 |
|  |  |  |  |  |  |  |  |
| *Other* |  |  |  |  |  |  |  |
| Cerebrovascular disorders in other diseases |  | I68 |  |  |  |  |  |
| Hypertensive encephalopathy |  | I67 |  |  |  |  | I67.4 |
| Occlusion/stenosis of arteries without infarct |  | I65, I66 |  |  |  |  |  |
| Other encephalopathy, cerebral compression, oedema |  | G93.49 |  |  |  |  | G93 |
| Other cerebrovascular diseases |  | I67 |  |  |  |  |  |
| Progressive multifocal leukoencephalopathy |  | A81.2 |  |  |  |  |  |
| Stroke not specified as haemorrhage or infarction |  |  | I64 | I64 | I64 |  |  |
|  |  |  |  |  |  |  |  |
| ***Psychosis*** |  |  |  |  |  |  |  |
| Puerperal psychosis |  |  | F53.1 | F53.1 | F53.1 |  |  |
| Brief psychotic episode |  |  | F23 | F23 | F23 |  |  |
|  |  |  |  |  |  |  |  |
| ***Status epilepticus*** |  |  |  |  |  |  |  |
| Status epilepticus (grand mal only) |  |  | G41 | G41 | G41 |  |  |
| Status epilepticus  (grand mal, petit mal, complex partial, other, unspecified) |  |  |  |  |  |  |  |
|  |  |  |  |  |  |  |  |
| **Haematological** |  |  |  |  |  |  |  |
| DIC |  | D65 | D65 | D65 | D65 |  | D65 |
| Other/unspecified coagulation defect |  | D68.8, D68.9 |  |  |  |  |  |
| Postpartum coagulation defects |  | O72.3 |  |  |  |  |  |
|  |  |  |  |  |  |  |  |
| Sickle cell disease with crisis |  |  |  |  |  |  |  |
| SCD with crisis (Hb-SS) |  | D57.0 | D57.0 | D57.0 | D57.0 |  |  |
| SCD with crisis (Sickle/Hb-C,  Sickle thalassaemia, other) |  | D57.21, D57.41,  D57.81 |  |  |  |  |  |
|  |  |  |  |  |  |  |  |
| Thromboembolism (and air embolism) |  |  |  |  |  |  |  |
| Pulmonary embolism (PE) with acute  cor pulmonale (septic, saddle, other) |  | I26.0 |  |  |  | I26.0 | I26 |
| PE without acute cor pulmonale  (septic, saddle, subsegmental, other) |  | I26.90, I26.92, I26.93,  I26.94, I26.99 |  |  |  | I26.9 |  |
| Air embolism, 1st trimester |  |  | O88.0 | O88.0 | O88.0 | O88.0 | O88.0 |
| Air embolism, 2nd trimester-puerperium  or unspecified |  | O88.012, O88.013, O88.019, O88.02, O88.03 |  |  |  |  |  |
| Thromboembolism, 1st trimester |  |  | O88.2 | O88.2 | O88.2 | O88.2 | O88.2 |
| Thromboembolism, 2nd trimester-puerperium  or unspecified |  | O88.212, O88.213,  O88.219, O88.22, O88.23 |  |  |  |  |  |
| Pyemic/septic embolism, 1st trimester |  |  | O88.3 | O88.3 | O88.3 | O88.3 | O88.3 |
| Pyemic/septic embolism, 2nd trimester-  puerperium or unspecified |  | O88.312, O88.313,  O88.319, O88.32, O88.33 |  |  |  |  |  |
| Other embolism, 1st trimester |  |  | O88.8 | O88.8 | O88.8 | O88.4 | O88.8 |
| Other embolism, 2nd trimester-  puerperium or unspecified |  | O88.812, O88.813,  O88.819, O88.82, O88.83 |  |  |  |  |  |
| Iatrogenic air embolism |  | T80.0XXA |  |  |  |  |  |
|  |  |  |  |  |  |  |  |
| Phlebitis and thrombophlebitis, lower limb |  |  |  |  |  | I80.1, I80.20, I80.29 |  |
| Portal vein, subclavian, vena cava,  other venous thrombosis |  |  |  |  |  | I81, I82.80, I82.88, I82.29 |  |
| Deep phlebothrombosis, puerperium |  |  |  |  |  | O87.1 |  |
|  |  |  |  |  |  |  |  |
| ***Hepatic*** |  |  |  |  |  |  |  |
| Toxic liver disease (drug-induced cholestasis/hepatitis/fibrosis/other/unspecified) |  |  |  |  | K71 |  |  |
| Hepatic failure |  |  |  |  | K72 |  | K72.0 |
|  |  |  |  |  |  |  |  |
| ***Renal*** |  |  |  |  |  |  |  |
| ARF/postpartum ARF |  | N17, O90.4 | N17, O90.4 | N17, O90.4, | N17, O90.4 |  | N17, O90.4 |
| Intraoperative/postprocedural complications/ disorders of the genitourinary system |  |  | N99.0 | N99.0 | N99.0 |  |  |
| Unspecified kidney failure |  |  | N19 | N19 | N19 |  |  |
|  |  |  |  |  |  |  |  |
| **HIV disease** |  |  |  |  |  |  |  |
| Symptomatic HIV/AIDS |  |  |  |  | B20, O98.7 |  |  |
| HIV leading to malignant neoplasms,  other diseases resulting from HIV, acute HIV |  |  |  |  | B21-24 |  |  |
|  |  |  |  |  |  |  |  |
| **Severe infection/septicaemia/sepsis** |  |  |  |  |  |  |  |
| Puerperal sepsis |  | O85 |  | O85 | O85, O75.3 | O85 |  |
| Severe sepsis (without/with septic shock) |  | R65.20, R65.20 |  |  |  |  |  |
| Sepsis or septic shock following a procedure/obstetric procedure |  | T81.44XA, T81.12XA, O86.04 |  |  |  |  |  |
| Septic arterial embolism |  | I76 |  |  |  |  |  |
| Sepsis (streptococcal, listerial, other) |  | A40, A41, A32.7 |  |  |  |  | A40, A41 |
| Sepsis (actinomycosis) |  |  |  |  |  |  | A42.7 |
| Disseminated candidiasis/candidal sepsis |  |  |  |  |  |  | B37.7 |
|  |  |  |  |  |  |  |  |
| **Shock** |  |  |  |  |  |  |  |
| Shock during/following labour and delivery |  | O75.1 | O75.1 | O75.1 | O75.1 | O75.1 |  |
| Shock (cardiogenic, hypovolaemic, septic, other, unspecified) |  | R57 | R57 | R57.0, R57.1, R57.2,  R57.8, R57.9 | R57 |  | R57 |
| Shock due to anaesthesia |  | T88.2XXA |  |  |  |  |  |
| Anaphylactic shock, unspecified, initial encounter |  | T78.2XXA |  |  |  |  |  |
| Shock folllowing a procedure |  | T81.10XA-.12XAT81.19XA |  |  |  |  |  |
| Anaphylactic reaction due to […]  administered medicament |  | T88.6XXA | T88.6 | T88.6 | T88.6 |  |  |
| Anaphylactic reaction due to serum |  |  | T80.5 | T80.5 | T80.5 |  |  |
|  |  |  |  |  |  |  |  |
| **PROCEDURAL, OBSTETRIC** |  |  |  |  |  |  |  |
| Curettage |  |  |  |  |  |  |  |
| Curettage of uterus + GA |  |  | ✔ | ✔ |  |  |  |
| Dilatation and curettage/aspiration/  evacuation + transfusion |  |  |  |  | ✔ |  |  |
|  |  |  |  |  |  |  |  |
| **Evacuation of haematoma** |  |  |  |  |  |  |  |
| Evacuation of vulval/vaginal haematoma |  |  |  | ✔ |  |  |  |
| Evacuation of incisional  haematoma + transfusion |  |  |  |  | ✔ (+transfusion) |  |  |
| Evacuation of incisional perineal haematoma |  |  | ✔ |  |  |  |  |
| Evacuation of non-incisional haematoma of perineum/vagina |  |  | ✔ |  |  |  |  |
| Evacuation of caesarean wound haematoma |  |  | ✔ |  |  |  |  |
| Aspiration of haematoma of organ |  |  |  | ✔ |  |  |  |
|  |  |  |  |  |  |  |  |
| Open drainage of abdominopelvic abscess |  |  | ✔ | ✔ |  |  |  |
| Image-guided drainage of  abdominopelvic abscess |  |  |  | ✔ |  |  |  |
|  |  |  |  |  |  |  |  |
| **Hysterectomy** |  |  |  |  |  |  |  |
| Abdominal hysterectomy |  | ✔ | ✔ | ✔ | ✔ | ✔ | ✔ |
| Vaginal hysterectomy |  |  |  |  |  |  |  |
| Abdominal hysterectomy (not bladder  neck or vaginal fixation/pelvic floor repair) |  |  |  |  | ✔ |  |  |
| Caesaren/peripartum hysterectomy |  |  |  |  | ✔ | ✔ | ✔ |
|  |  |  |  |  |  |  |  |
| **Abdominal re-intervention** |  |  |  |  |  |  |  |
| Reclosure of postoperative disruption of  abdominal wound |  |  | ✔ | ✔ |  |  |  |
| Re-exploration of abdominal surgical site  +/- arrest of postoperative surgical bleeding |  |  |  | ✔ |  |  |  |
| Re-opening of abdomen |  |  | ✔ | ✔ |  | ✔ |  |
| Opening of abdomen and exploration of groin |  |  |  | ✔ |  |  |  |
| Secondary suture of skin, resuture of skin |  |  |  | ✔ |  | ✔ |  |
| Refashioning of scar |  |  |  | ✔ |  |  |  |
| Control of postoperative bleeding/thrombosis  after intraabdominal vascular procedure |  |  | ✔ |  |  |  |  |
| Control of postoperative haemorrhage  folllowing gynaecological surgery |  |  | ✔ |  |  | ✔ |  |
| Reoperation for deep infection in  obstetric surgery, laparotomy and  drainage of peritoneal cavity |  |  |  |  |  | ✔ |  |
| Reoperation for insufficiency of  anastomosis or suture in  gynaecological surgery |  |  |  |  |  | ✔ |  |
|  |  |  |  |  |  |  |  |
| **Repair of abdominopelvic organs** |  |  |  |  |  |  |  |
| Repair of obstetric laceration to bladder/  urethra without perineal involvement |  |  | ✔ |  | ✔ |  |  |
| Repair of ruptured bladder |  |  | ✔ | ✔ |  |  |  |
| Repair of rupture of urethra |  |  |  | ✔ |  |  |  |
|  |  |  |  |  |  |  |  |
| Repair of ruptured uterus |  |  | ✔ |  |  |  | ✔ |
| Surgical correction of inverted uterus |  |  | ✔ |  |  |  |  |
| Surgical repair of laceration uterus  + transfusion |  |  |  |  | [✔ + transfusion](http://5.pc.80.jm/) |  |  |
|  |  |  |  |  |  |  |  |
| **Surgical management of haemorrhage** |  |  |  |  |  |  |  |
| Embolisation of artery |  |  | ✔ | ✔ | ✔ | ✔ |  |
| Open embolisation |  |  |  | ✔ |  |  |  |
| Ligation of artery |  |  | ✔ | ✔ | ✔ | ✔ |  |
| Ligation of vein |  |  |  | ✔ |  |  |  |
| Transluminal procedures of vein |  |  |  | ✔ | ✔ |  |  |
| Compression suture of uterus |  |  |  |  | ✔ |  |  |
| Balloon compression of uterus/tamponade |  |  |  |  | ✔ | ✔ |  |
|  |  |  |  |  |  |  |  |
| **PROCEDURAL (AND TREATMENT-BASED ICD CODES), NON-SPECIFIC** |  |  |  |  |  |  |  |
|  |  |  |  |  |  |  |  |
| Exploratory laparotomy |  |  |  |  |  | ✔ | ✔ |
| Exploratory laparoscopy |  |  |  |  |  | ✔ |  |
| Cystotomy |  |  | ✔ | ✔ |  |  |  |
| Repair of fistula |  |  |  | ✔ |  |  |  |
|  |  |  |  |  |  |  |  |
| Bowel resection and anastomosis  (jejunectomy, ileectomy) |  |  | ✔ | ✔ |  |  |  |
| Resection of small intestine with formation of stoma |  |  | ✔ |  |  |  |  |
| Other procedures involving ileum |  |  |  | ✔ |  |  |  |
| Colectomy and anastomosis/exteriorisation/ileostomy |  |  | ✔ | ✔ |  |  |  |
| Endoscopic procedures on lesion of distal bowel/sigmoid |  |  |  | ✔ |  |  |  |
| Excision of rectum |  |  |  | ✔ |  |  |  |
| Repair of mesentery of small intestine/colon |  |  |  | ✔ |  |  |  |
| Endoscopic resection of lesion of peritoneum |  |  |  | ✔ |  |  |  |
| Formation of ileostomy reservoir |  |  | ✔ |  |  |  |  |
| Suture of small/large intestine |  |  | ✔ |  | ✔ |  |  |
| Other repair (small/large intestine) |  |  | ✔ |  | ✔ |  |  |
|  |  |  |  |  |  |  |  |
| ***Cardiovascular*** |  |  |  |  |  |  |  |
| Restoration of cardiac rhythm |  | ✔ |  |  |  |  |  |
| Performance of cardiac output |  | ✔ |  |  |  |  |  |
|  |  |  |  |  |  |  |  |
| ***Transfusion (in isolation)*** |  |  |  |  |  |  |  |
| Whole blood or packed cells |  | ✔ | ✔ |  | With diagnostic codes |  |  |
| Platelets |  |  |  |  |  |  |  |
| Coagulation factors or other serum |  |  |  |  |  |  |  |
| Exchange transfusion |  |  | ✔ |  |  |  |  |
|  |  |  |  |  |  |  |  |
| ***Intensive treatment unit*** |  |  |  |  |  |  |  |
|  |  |  |  |  |  |  |  |
| ***Ventilation*** |  |  |  |  |  |  |  |
| Tracheostomy |  | ✔ | ✔ | ✔ | ✔ |  |  |
| Cricothyroidostomy |  |  |  | ✔ |  |  |  |
| Exteriorisation of trachea |  |  |  | ✔ |  |  |  |
| Tracheostomy-related |  |  | ✔ |  |  |  |  |
| Continuous ventilation |  | ✔ | ✔ |  |  |  |  |
| Non-invasive ventilation |  |  | ✔ | ✔ |  |  |  |
| Invasive ventilation |  |  |  | ✔ | ✔ |  |  |
|  |  |  |  |  |  |  |  |
| ***Renal (dialysis)*** |  |  |  |  |  |  |  |
| Haemodialysis |  |  | ✔ | ✔ | ✔ |  |  |
| Dialysis (all) |  |  |  |  |  |  |  |
| Insertion of peritoneal dialysis catheter |  |  | ✔ | ✔ |  |  |  |
| Replacement/removal of  peritoneal dialysis catheter |  |  | ✔ |  |  |  |  |
